# Shaping the subway microbiome through probiotic-based sanitation during the COVID-19 emergency: a pre–post case–control study

**DOI:** 10.1186/s40168-023-01512-2

**Published:** 2023-03-30

**Authors:** Maria D’Accolti, Irene Soffritti, Francesca Bini, Eleonora Mazziga, Carolina Cason, Manola Comar, Antonella Volta, Matteo Bisi, Daniele Fumagalli, Sante Mazzacane, Elisabetta Caselli

**Affiliations:** 1 Section of Microbiology, Department of Chemical, Pharmaceutical and Agricultural Sciences, and LTTA, University of Ferrara, 44121 Ferrara, Italy; 2 CIAS Research Center, University of Ferrara, 44122 Ferrara, Italy; 3 Department of Advanced Translational Microbiology, Institute for Maternal and Child Health, IRCCS “Burlo Garofolo”, Trieste, Italy; 4 Department of Medical Sciences, University of Trieste, Trieste, Italy; 5 Facility Management Unit, Azienda Trasporti Milanesi S.P.A, 20121 Milan, Italy

**Keywords:** Subway microbiome, Probiotic sanitation, Disinfection, COVID-19

## Abstract

**Background:**

The COVID-19 pandemic has highlighted the extent to which the public transportation environment, such as in subways, may be important for the transmission of potential pathogenic microbes among humans, with the possibility of rapidly impacting large numbers of people. For these reasons, sanitation procedures, including massive use of chemical disinfection, were mandatorily introduced during the emergency and remain in place. However, most chemical disinfectants have temporary action and a high environmental impact, potentially enhancing antimicrobial resistance (AMR) of the treated microbes. By contrast, a biological and eco-sustainable probiotic-based sanitation (PBS) procedure was recently shown to stably shape the microbiome of treated environments, providing effective and long-term control of pathogens and AMR spread in addition to activity against SARS-CoV-2, the causative agent of COVID-19. Our study aims to assess the applicability and impact of PBS compared with chemical disinfectants based on their effects on the surface microbiome of a subway environment.

**Results:**

The train microbiome was characterized by both culture-based and culture-independent molecular methods, including 16S rRNA NGS and real-time qPCR microarray, for profiling the train bacteriome and its resistome and to identify and quantify specific human pathogens. SARS-CoV-2 presence was also assessed in parallel using digital droplet PCR. The results showed a clear and significant decrease in bacterial and fungal pathogens (*p* < 0.001) as well as of SARS-CoV-2 presence (*p* < 0.01), in the PBS-treated train compared with the chemically disinfected control train. In addition, NGS profiling evidenced diverse clusters in the population of air vs. surface while demonstrating the specific action of PBS against pathogens rather than the entire train bacteriome.

**Conclusions:**

The data presented here provide the first direct assessment of the impact of different sanitation procedures on the subway microbiome, allowing a better understanding of its composition and dynamics and showing that a biological sanitation approach may be highly effective in counteracting pathogens and AMR spread in our increasingly urbanized and interconnected environment.

Video Abstract

**Supplementary Information:**

The online version contains supplementary material available at 10.1186/s40168-023-01512-2.

## Background

The human body is host to trillions of microbes including bacteria, fungi, and viruses. They influence our health and disease and are, in turn, influenced by the environment. At the same time, the environment itself hosts an extensive assortment of microbes to which we are exposed every day. The built environment (BE) has become the primary habitat for modern humans, including not only homes, community buildings, and hospitals, but also transport [1–3]. Mass transport environments, including of subways, can favor the continuous flow and exchange of microbes among humans and among different BEs, potentially leading to a very rapid pathogen spread and impacting high numbers of individuals [4, 5]. The subway surface microbiome has recently been characterized using deep sequencing techniques, providing a comprehensive picture of the bacterial component and of its antimicrobial resistance (AMR) features in numerous geographical regions around the world [6–10]. In particular, the International Metagenomics and Metadesign of Subways and Urban Biomes (MetaSUB) consortium analyzed almost 5000 samples from 60 cities around the world [10], providing a worldwide atlas of the subway microbial community and showing the presence of over 4000 known microbial species, including bacteria, archaea, and virus species [10], and finally confirming the potential role of the urban transit systems in microbe transmission, serving as a daily contact interface for billions of urban inhabitants.

The COVID-19 pandemic has further highlighted the possible role of mass transport in virus transmission given, on the one hand, the features of resistance demonstrated by SARS-CoV-2 in the external environment [11, 12] and, on the other hand, the identification of a large number of viral sequences with potential viral–host interactions in the transport environment [10].

In accordance with these observations, the mandatory use of chemical disinfectants has been introduced on a massive scale worldwide, even being applied in non-healthcare settings such as public transport [13–15], to counteract the spread of SARS-CoV-2, and they are still being applied.

However, the action of disinfectants is temporary, permitting rapid recontamination of the treated surfaces [16], and, thus, not necessarily protecting people from the contact with potential pathogens. Furthermore, chemical disinfectants have a high environmental impact, and their widespread use can result in elevated levels of pollution in water and earth [17]. Last, super-sanitation can deeply affect the environmental microbiome, potentially leading to increased microbial resistance against not only disinfectants but also antimicrobial drugs [18, 19]. Due to these limitations, which may exacerbate the indicated problems, we recently studied a low-impact method aimed at long-term sanitation of the environment without increasing undesirable side effects on microbes, people, or the environment. Such a method, namely probiotic-based sanitation (PCHS, Probiotic Cleaning Hygiene System), relies on the use of a mild eco-friendly detergent containing spores of selected probiotics, belonging to the *Bacillus* genus, that are able to colonize treated surfaces and displace surrounding pathogens via a mechanism of competitive exclusion [20]. PCHS, compared with chemical disinfection, was shown to induce in the hospital environment a stable abatement of pathogens (80% more than chemical disinfectants), a 3-log decrease in AMR of the residual population, and a concomitant 52% decrease in healthcare-associated infections (HAIs) [16, 18, 20–26]. This system was also shown to induce long-lasting decontamination from enveloped viruses in vitro [16] and was thus successfully used in the emergency ward of a pediatric hospital during the COVID-19 pandemic [27], based on its demonstrated stability and safety of use [22].

Based on these premises, the aim of the present study was to assess the applicability and the effectiveness of PCHS for sanitation purposes in the subway of a big city in Northern Italy.

## Methods

### Aim and setting of the study

The study aimed to compare the impact of biological (PCHS) vs. chemical sanitation on the subway microbiome composition, with particular regard to human pathogens (including SARS-CoV-2) and associated AMR. To this purpose, the study was performed in collaboration with the Milan Transport Company (Azienda Trasporti Milanesi, ATM; Milan, Italy) after approval by the technical scientific committee of the company. Two underground driverless trains with superimposable characteristics were enrolled in the study. Each had four compartments corresponding to a total of 96 seats and 438 standing places for a total 50.5 m in length and 73 m^2^ of surface.

At the time of the study, the ATM sanitation protocol, as proposed by the cleaning company in compliance with current Italian regulations for COVID-19 pandemic management, included the use of ethanol-, chlorine-, and ammonium salt-based products. To compare the effectiveness of chemical vs. PCHS sanitation, one train continued to receive the routine chemical disinfection and was used as a control, whereas a PCHS sanitation protocol (Probiotic Cleaning Hygiene System; PCHS®, Copma scrl, Ferrara, Italy) was implemented in the other train. In compliance with the current national COVID-19 guidelines for mass transport hygienization, which mandatorily indicated to include chemical disinfectants, it was not possible to completely replace chemical disinfection with PCHS. Thus, based on preliminary compatibility tests, PCHS sanitation was used to replace chlorine disinfection, whereas ethanol and ammonium disinfectants were maintained throughout the study. PCHS sanitation, as chemical disinfectants, was applied by using prepreg cloths and nebulization, maintaining the same time schedule used for chemical disinfectants. Based on compatibility assays, PCHS was applied 30 min after alcohol/ammonium disinfection to preserve PCHS probiotics viability.

The study period lasted for a total of 12 weeks from September to December 2021.

During the whole study period, data were collected on the train bioburden and passenger flows in both trains for determining the correlations between the contamination level, number of travelers, and type of applied sanitation.

### Compatibility tests in vitro

At the time of the study, the use of chemical disinfectants for mass transport sanitation was mandatory due to the COVID-19 emergency and had to comply with the Italian Ministry of Health directives, and because PCHS is based on the presence of active probiotic bacteria, the compatibility between PCHS and each disinfectant included in the ATM protocol was assessed prior to starting the field study. Briefly, the viability of the PCHS-derived *Bacillus* (hereafter PCHS-*Bacillus*) was measured following exposure to chlorine-, ethanol-, and ammonium-based products on hard nonporous surfaces under controlled laboratory conditions. Two types of assays were performed to separately assess (1) the viability of PCHS-*Bacillus* seeded on surfaces subsequently treated with disinfectants and (2) the viability of PCHS-*Bacillus* seeded on surfaces previously treated with disinfectants. The latter was performed using both spores and germinated vegetative forms of PCHS-*Bacillus*.

In the first assay, the PCHS detergent, containing 10^7^ *Bacillu*s spores/mL, was diluted 1:100 in sterile water, then 10 μL of diluted detergent (corresponding to 10^3^ spores) was seeded on a 24 cm^2^ surface and left to dry. Chemical disinfectants were prepared according to the manufacturer’s instructions, uniformly applied by nebulization on the PCHS-treated surfaces and left to act for the times indicated by the manufacturer, which were 1 or 15 min for chlorine/ethanol-based or ammonium products, respectively. At the end of the action time, the residual PCHS-*Bacillus* was collected onto 24 cm^2^ contact plates (55 mm diameter Replicate Organism Detection and Counting, RODAC) containing tryptic soy agar (TSA, Sharlab, Milan, Italy) as a nonselective general medium. RODAC plates were incubated for 24 h at 37 °C, then the number of *Bacillus* colony forming units (CFU) was counted.

In the second assay type, the disinfectants were uniformly spread by nebulization on surfaces and left to dry. After 0.5, 1, 2, 3, 4, and 24 h from disinfectant application, 10 μL of diluted PCHS detergent (10^3^ spores) were spread on a 24 cm^2^ area of the pre-disinfected surface. After 1 or 15 min of contact time (for chlorine/ethanol- or ammonium-based disinfectants, respectively), the amount of PCHS-*Bacillus* was measured using TSA RODAC sampling and CFU counting after 24 h incubation at 37 °C. This assay was also performed using the PCHS-*Bacillus* germinated spores, i.e., the bacterial vegetative forms. Here, the PCHS-*Bacillus* spores were first allowed to germinate and grow in tryptic soy broth (TSB, Biolife, Monza, Italy) at 37 °C overnight, then the bacterial suspension was diluted to obtain a final concentration of 10^5^ CFU/mL and 10 μL (corresponding to 10^3^ bacteria) was spread on the pre-disinfected surfaces. Following 1–15 min of contact time, *Bacillus* CFU were counted after TSA RODAC collection and 24 h incubation. In each assay, sterile water and a sporicidal disinfectant (Viroxid Spray®, IDS SpA, Savona, Italy) were used as negative and positive controls, respectively.

### Sanitation procedures on field

Both chemical and PCHS sanitation were performed by the same cleaning company, specialized in environmental sanitation and adequately trained for both disinfectants and PCHS application (©Fulgens Italia S.R.L, Italy). Chemical disinfection was performed in compliance with current regulations and according to the manufacturer’s instructions and included (i) daily cleaning of seats, handrails, doors, and floors with alcohol-based disinfectant (KEM Alcohol Duo, containing 78% ethanol; Kemika SpA, Alessandria, Italy) and chlorine-based disinfectant (Biospot, containing 200 ppm of active chlorine; Kemika SpA, Alessandria, Italy); (ii) weekly treatment with nebulization of a quaternary ammonium salt-based product (Hygiene Spray Professional, containing benzalkonium chloride and O-phenylphenol; GEN-ART srl, Lanuvio, Rome, Italy); and iii) bimonthly thorough disinfection with alcohol- and chlorine-based products.

PCHS sanitation was applied using both prepreg cloths and nebulization [24, 25, 28], similarly to the chemical disinfectants, and without altering the time schedule of the cleaning interventions or the type of treated surfaces in the two enrolled trains. PCHS sanitation was applied instead of chlorine-based disinfection, whereas ethanol and ammonium disinfectants were continuously applied in compliance with the national COVID-19 guidelines. Based on compatibility assay results, PCHS was applied 30 min after alcohol- or ammonium-based disinfection.

All sanitation procedures were performed in the absence of people, at the end of the daily train run.

### Environmental sampling

Six sampling campaigns were performed in both enrolled trains, at the following times: T0 (before PCHS implementation), and T1, T2, T3, T4, and T5, respectively 2, 4, 6, 9, and 12 weeks after PCHS introduction in the PCHS-train. Samplings were performed biweekly except for the last timepoint (T5), which was performed after three weeks due to unavailability of ATM personnel to support sampling technicians during early December holiday period. Sampling was performed late at night, at the end of the train run, and before applying the cleaning protocols. Twelve points were sampled at each timepoint, corresponding to different areas of the train: floors (3 samples), seats (3 samples), handrails (2 samples), doors (2 samples), and air filters (2 samples). Air filters were not available at T3 and T5 timepoints. The same points were simultaneously sampled using two different methods according to subsequent microbiological or molecular analyses. All samples were immediately refrigerated at 4 °C and transported to the laboratory within 24 h.

For conventional microbiological analyses (CFU count), each point was sampled in duplicate as previously described [24, 25], by using RODAC contact plates containing the following specific culture media: TSA (Sharlab, Milan, Italy) for the total bacteria count; Baird Parker agar (Sharlab, Milan, Italy) for *Staphylococcus* spp. detection and *Bacillus* spp. count; MacConkey agar (Sharlab, Milan, Italy), selective for *Enterobacteriaceae* spp.; cetrimide agar (Sharlab, Milan, Italy) selective for *Pseudomonas* spp.; *Clostridium difficile* selective agar for *Clostridium difficile* growth (Lickson, Palermo, Italy); and Sabouraud dextrose agar (Liofilchem, Millipore, Milan, Italy) selective for mycetes, including *Candida* and *Aspergillus* genera. Ready-to-use RODAC plates of 55 mm diameter (corresponding to 24 cm^2^ surface) were applied to surfaces for 10 s by using a contact weight applicator (VWR International Srl, Milan, Italy).

For molecular analyses, the same twelve points were sampled by using sterile rayon swabs, rubbed onto a 100 cm^2^ area, as previously described [24, 25, 28]. Each point was sampled in duplicate by using swabs pre-moistened in sterile TSB broth (Biolife, Monza, Italy) or phosphate-buffered saline (PBS), which were then used for different molecular assays.

### Microbiological analyses

The RODAC plates derived from sampling train surfaces and air filters were incubated in appropriate conditions for bacteria and mycetes detection. Briefly, the incubation was performed at 37 °C for 24 or 48 h, for general and selective bacterial media, respectively. Plates specific for mycetes were incubated at 25 °C for 72 h. *Clostridium* spp. agar plates were anaerobically incubated at 37 °C for 48 h in anaerobic jars (Anaerogen Systems, ThermoFisher Scientific Inc). At the end of incubation, the microbial growth was determined by CFU enumeration. For each timepoint, 240 samples from surfaces and 48 samples from air filters were collected in total from the two trains, except for two timepoints (T3 and T5) when the air filters were not available and therefore 240 samples were collected. Overall, a total of 1632 samples were collected and analyzed during the study.

### Molecular analyses

The swabs pre-wet in TSB or PBS, used to sample train surfaces and air filters, were processed differently.

TSB swabs were used for resistome analysis, that was performed by microarray. Briefly, immediately after rubbing, swabs were placed in 5 mL TSB and incubated at 37 °C for 24 h. The grown microbial population was collected by centrifugation at 12,000* g* at 4 °C and frozen at − 80 °C until use [24, 25]. The total DNA was extracted from frozen pellets using the Exgene Cell SV mini kit (Gene All, South Korea), and 1 µg of DNA was analyzed using the Microbial DNA qPCR Array for Antibiotic Resistance Genes (Qiagen, Germany), providing the simultaneous detection and quantification of 84 antibiotic resistance genes, as previously described [23–25].

PBS swabs were used for SARS-CoV-2 analysis and next-generation sequencing (NGS) analyses. To these purposes, immediately after rubbing, swabs were placed in 0.4 mL PBS and directly frozen at − 80 °C until use, as previously described [29]. Then, total nucleic acids (TNA) were then extracted from the samples using the Maxwell CSC platform equipped with the HT Viral TNA Kit (Promega, Milan, Italy) [30]. For SARS-CoV-2 detection and quantification, 50 ng of TNA were analyzed for the presence SARS-CoV-2 by droplet digital PCR (ddPCR), using the SARS-CoV-2 ddPCR Kit (Bio-Rad Laboratories, Milan, Italy) [30].

For NGS analysis, the extracted TNA were analyzed to profile the whole bacteriome of the enrolled trains. Based on the level of contamination detected by CFU count in collected samples, only the specimens from floors and air filters were analyzed by NGS, as representative of surface and air microbiomes. Briefly, 100 ng of TNA were characterized by sequencing the V3 region of the 16S rRNA as previously described [27, 31], including no DNA template as a negative control. First, quantitative real-time PCR (qPCR) was performed using degenerate primers, then a nested PCR targeting the V3 region of the 16S rRNA gene was performed as previously described [32]. The obtained sequences were processed using Quantitative Insights Into Microbial Ecology (QIIME 2 2020.2) [32]. Taxonomy assignment was performed against the reference taxonomy database SILVA V.132 with a similarity threshold of 97%.

Overall, 136 TSB-swabs and 136 PBS-swabs were collected from each train (120 from surfaces and 16 from air filters), for a total 272 samples collected and analyzed.

### Statistical analyses

Statistical analyses were performed using GraphPad Prism software. Parametric and nonparametric Student’s *t* test, Mann–Whitney, and ANOVA tests were used to compare groups, assuming a *p* value ≤ 0.05 to indicate a statistically significant difference. To analyze the microarray data, Bonferroni correction for multiple comparisons was applied to the value detected in Student’s *t* test (a *p*_*c*_ value ≤ 0.05 was considered significant). Statistical analyses for NGS results were performed with QIIME 2 (2020.2). Beta diversity was assessed using a Bray–Curtis distance matrix and used in principal coordinates analysis (PCoA). The permutational multivariate analysis of variance (PERMANOVA) test was performed for comparison of groups.

## Results

### Compatibility between PCHS and disinfectants

At the time of the study, the sanitation protocol used for mass transport, aimed at addressing the COVID-19 emergency, imposed the mandatory use of chemical disinfection as specified in the “Technical Specifications and Extraordinary Hygiene measures for COVID-19 pandemic,” defined by ATM in accordance with the directives of the Italian Ministry of Health [15]. Accordingly, ATM protocols for train sanitation included the daily use of alcohol and chlorine and a weekly treatment with ammonium-based products. Because the Probiotic Cleaning Hygiene System (PCHS) sanitation is based on competitive exclusion with the living probiotics included in the cleanser, preliminary compatibility assays were performed to assess the viability of the PCHS- *Bacillus* in the presence of the chemical disinfectants imposed by the ATM disinfection protocol. Two assay types were performed to separately assess the viability of PCHS-*Bacillus* on surfaces subsequently or previously treated with each of the used disinfectants.

**Fig. 1:**
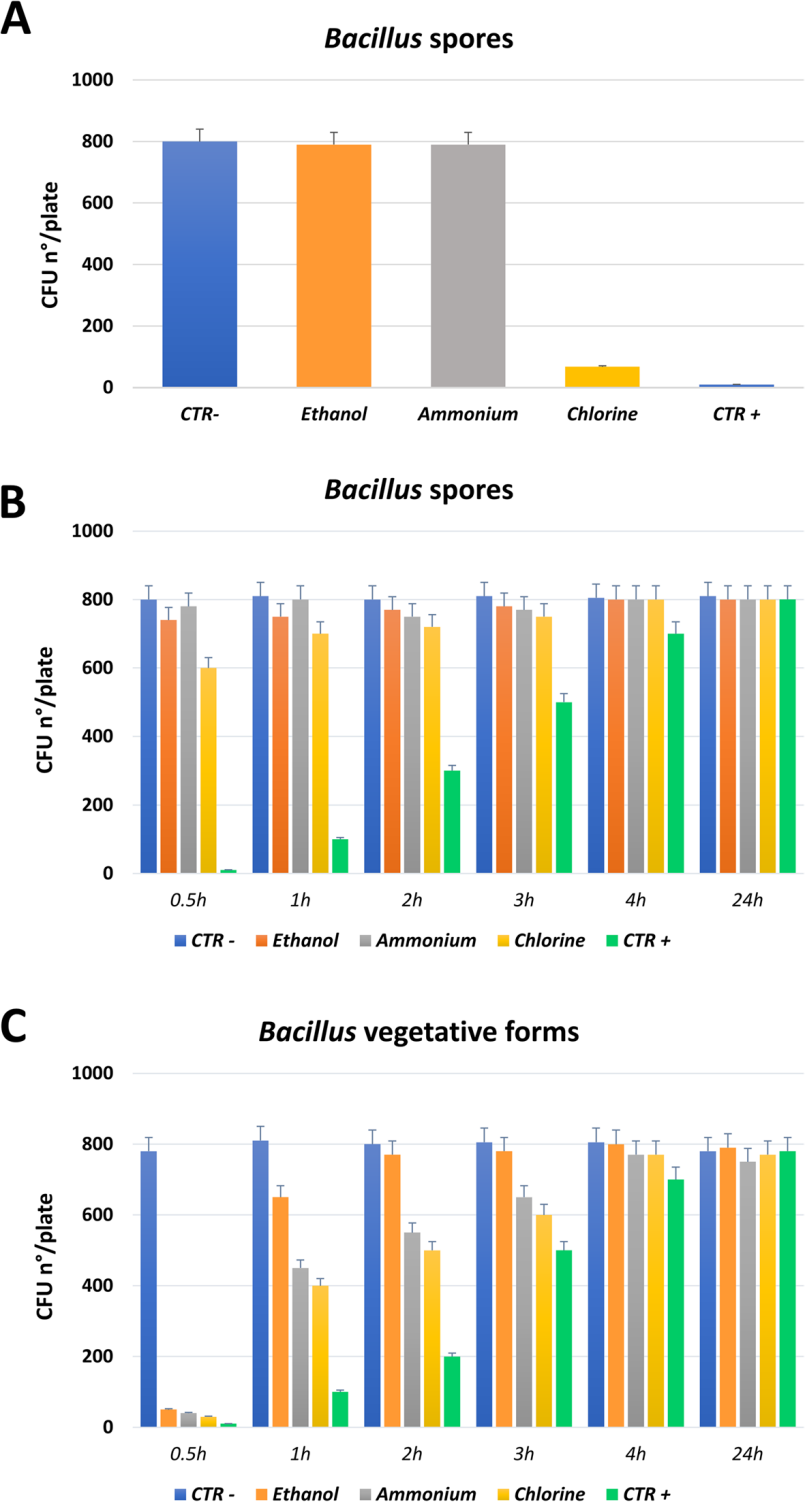
Tests of PCHS-*Bacillus* and disinfectant compatibility. PCHS-*Bacillus* viability in the presence of the indicated disinfectants was assessed in vitro on hard nonporous surfaces. **a** PCHS-*Bacillus* spores spread on surfaces and subsequently treated with the indicated disinfectants. **b** PCHS-*Bacillus* spores spread on surfaces previously treated with the indicated disinfectants. **c** PCHS-*Bacillus* growing bacteria seeded on surfaces previously treated with the indicated disinfectants. All results are expressed as mean CFU ± S.D. of duplicate samples in two independent experiments, measured after 24 h incubation on TSA plates

The results showed, as expected, that the chlorine-based product was effective in inactivating > 90% of *Bacillus* spores (> 90%) on surfaces, whereas no significant spore reduction (< 10%) was observed when alcohol- or ammonium-based products were applied after PCHS use (Fig. 1a). Likewise, no significant reduction in *Bacillus* spores (< 10%) was observed when PCHS was applied 1 h after previous disinfection with chlorine-, ethanol-, or ammonium-based products (Fig. 1b), whereas a significant reduction (> 90%) was observed when *Bacillus* vegetative forms, instead of spores, were spread on surfaces within 1 h from disinfectant application (Fig. 1c). At 30 min after application, disinfectants were inactive on spores except for chlorine (causing a 25% reduction of spore titer), whereas all disinfectants were still active against the vegetative form of PCHS-*Bacillus* (Fig. 1b-c). Thus, based on the results of the compatibility assays and in consideration of the PCHS-*Bacillus* germination time, the experimental sanitation protocol applied included the use of PCHS as a substitute for chlorine-based disinfection, and it was applied 30 min after disinfection with the alcohol- or ammonium-based disinfectants.

### Study set-up in subway

The study aimed to compare the impact of PCHS sanitation vs. chemical chlorine-based sanitation on train microbiome. Toward this purpose, two driverless trains of the Milan Transport Company (Azienda Trasporti Milano, ATM; Milan, Italy), with superimposable features, were enrolled in a 12-week study during the COVID-19 pandemic (September–December 2021). Due to the current anti-COVID-19 measures for mass transport sanitation, indicating mandatorily the use of chemical disinfection, it was not possible to completely replace chemical disinfectants with PCHS at the time of the study. Thus, to ensure compliance with the Italian directives and based on the compatibility assays results, the train that served as a control continued to receive routine chemical disinfection (chlorine, ethanol, ammonium) during the whole study period, whereas in the PCHS-treated train, PCHS sanitation replaced chlorine disinfection but ethanol- and ammonium-based disinfection was maintained.

**Fig. 2:**
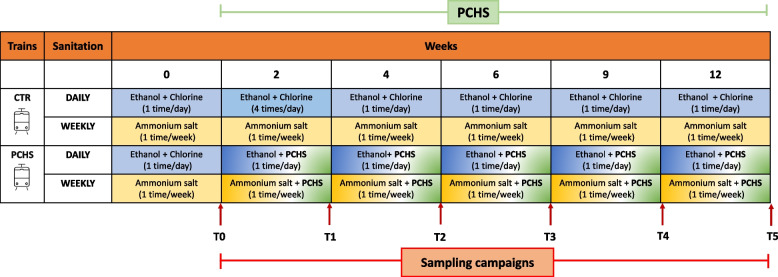
Schematic representation of the study design. Of the two trains enrolled in the study, the control train (CTR) continued to receive the chemical disinfection for the whole study period (12 weeks), whereas in the PCHS train the chlorine-based disinfection was replaced by PCHS sanitation. Daily and weekly sanitation protocols are indicated by different colors: light blue (ethanol/chlorine products, daily), dark blue (ethanol/chlorine products, 4 times/day), yellow (ammonium salt product, weekly), and green (PCHS introduction: ethanol/PCHS, daily, and PCHS weekly). Sampling campaigns at T0–T5 times are indicated by red arrows

More precisely, as schematized in Fig. 2, the control train received daily disinfection with ethanol and chlorine and weekly disinfection with ammonium nebulization, while the PCHS train received daily ethanol/PCHS sanitation and weekly ammonium/PCHS nebulization. Based on preliminary compatibility analysis, PCHS was applied 30 min after ethanol or ammonium disinfectants. All sanitation procedures were simultaneously performed in both trains in the absence of people.

Six sampling campaigns were performed to characterize the microbial surface and air contamination on both trains, before (T0) and after PCHS implementation, and at 2 (T1), 4 (T2), 6 (T3), 9 (T4), and 12 (T5) weeks (Fig. 2). At each timepoint, twelve total points were sampled in duplicate from surfaces (floors, seats, handrails, and doors) and air filters (not available at T3 and T5). Each sampled point was collected by both RODAC contact plates and sterile swabs, respectively, for subsequent microbiological and molecular analyses.

Passenger flow data were also collected during the whole study period, with no significant differences found between the control and PCHS trains (Table 1), despite minimal higher flows in the PCHS compared with the control train in all monitored periods.

**Table 1:** Passenger flow data for control and PCHS trains (^a^)

*Train*	*Time periods*				
	**T0-T1**	**T1-T2**	**T2-T3**	**T3-T4**	**T4-T5**
**CTR**	77,631	72,799	80,070	120,743	117,277
**PCHS**	78,074 (+ 0.57%)	84,644 (+ 16.27%)	83,132 (+ 3.82%)	126,333 (+ 4.63%)	126,284 (+ 7.68%)

^a^Data are reported as total number of passengers in the indicated time-period. The percentage difference in PCHS-train vs. control train are also reported in parentheses

### Microbial monitoring

Conventional microbial monitoring of train surfaces and air filters included determination of six groups of potential human pathogens, comprising bacteria (*Staphylococcus* spp., *Enterobacteriaceae* spp., *Pseudomonas* spp., and *Clostridium difficile*) and fungi (*Candida* and *Aspergillus* spp.), through RODAC plate sampling and CFU counting after appropriate incubation. Overall, 1632 samples were collected and analyzed.

**Fig. 3:**
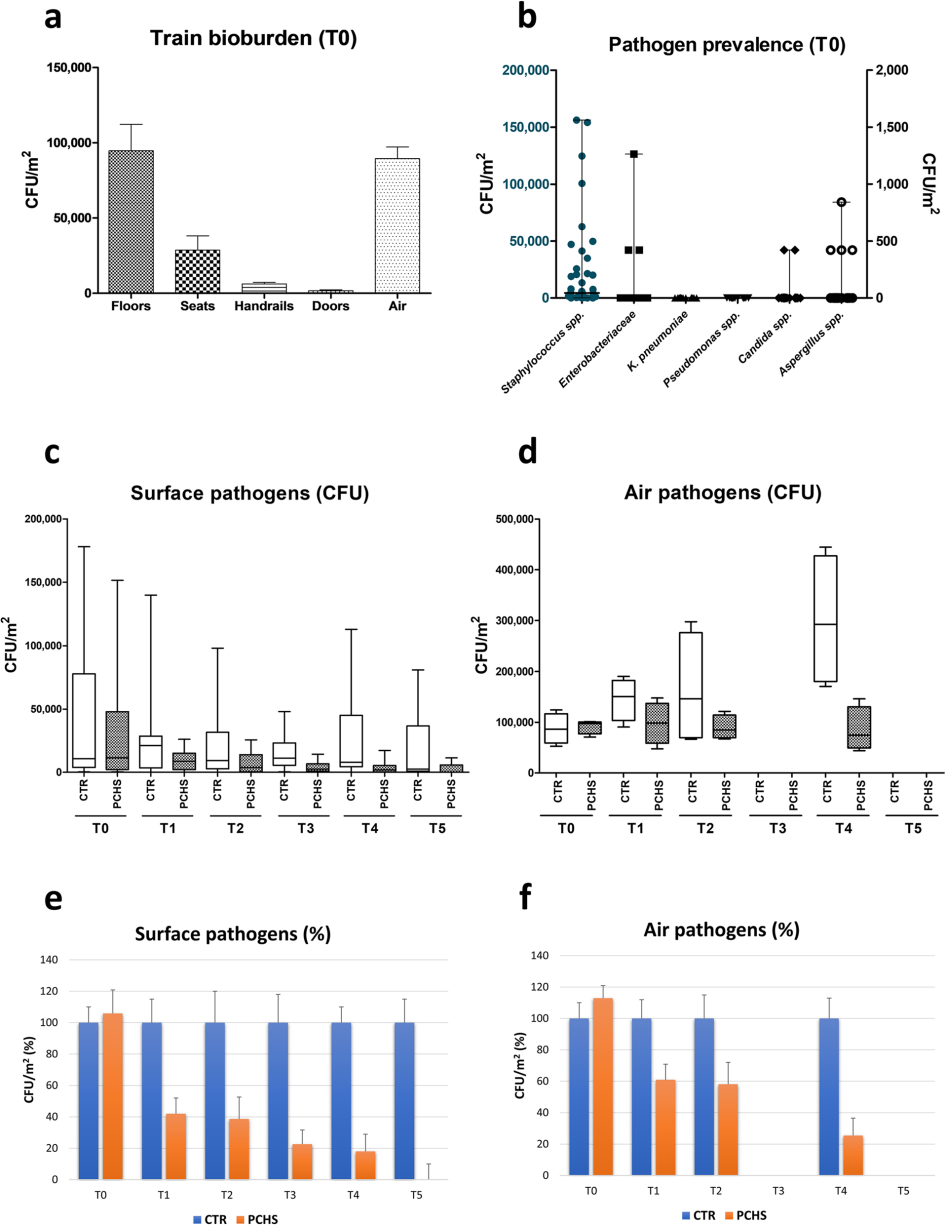
Pathogen contamination in enrolled trains. Surfaces (floor, door, handrails, and seats) and air filters were sampled using RODAC plates. Assayed pathogens included *Staphylococcus* spp., *Enterobacteriaceae* spp., *Pseudomonas* spp., *Clostridium* spp., *Candida* spp., and *Aspergillus* spp. **a** Contamination levels in tested surface (floors, seats, handrails, and doors) and air filter samples. Results are expressed as median values of CFU/m^2^ ± S.D. **b** Prevalence of assayed pathogens in total surface and air samples. Results are expressed as median values of CFU/m^2^ ± S.D. Values on the left Y axis refer to *Staphylococcus* spp., whereas values on the right *Y*-axis refer to the other microbes. **c**,** d** Pathogenic contamination levels before (T0) and after (T1–T5) the introduction of PCHS sanitation on surfaces (**c**) and air filters (**d**). The results are expressed as CFU/m^2^: median values (lower part of the box) and Q3 values (upper part of the box, representing the 75% percentile values) are shown, together with min and max values. **e**, **f** Comparison between the median levels of contamination detected in the control train (CTR), normalized to 100%, and those detected in the PCHS-train, expressed as percentage values of PCHS train versus the number of CFU/m^2^ detected in the control train, taken as 100%

The results obtained at T0 in both trains showed a very similar level of pathogenic contamination, expressed as the sum of the assayed pathogens in all tested surfaces, corresponding to 10,737 CFU/m^2^ (median value, range 421–178,105 CFU/m^2^) in the control train and 11,368 CFU/m^2^ (median value, range 421–69,895 CFU/m^2^) in the train assigned for PCHS treatment. However, the different sampled surfaces exhibited very different levels of pathogenic contamination, with significantly more contamination on the floors compared with seats, handrails, and doors (*p* < 0.001) (Fig. 3a). Specifically, pathogens amounted to 96,631 CFU/m^2^ (median value; range 18,105–189,057 CFU/m^2^) on floors, 19,368 CFU/m^2^ (median value; range 4211–43,368 CFU/m^2^) on seats, 5894 CFU/m^2^ (median value; range 2947–10,105 CFU/m^2^) on handrails, and 1739 CFU/m^2^ (median value; range 0–5474 CFU/m^2^) on doors. Air filters also showed high level of contamination, evidencing a total pathogenic load corresponding to 86,316 CFU/m^2^ (median value, range 52,632–124,211 CFU/m^2^) and 97,474 CFU/m^2^ (median value, range 70,737–101,053 CFU/m^2^) in the control and PCHS train, respectively.

At T0, the contamination was mainly attributable to coagulase-negative *Staphylococcus* spp., representing up to 56% of the total detected pathogens (median value 4526 CFU/m^2^, range 0–156,211 CFU/m^2^) (Fig. 3b). Gram-negative *Enterobacteriaceae* family was less abundant (median value 0 CFU/m^2^, range 0–1263 CFU/ m^2^), and other bacterial genera, including *Pseudomonas* spp.,* Clostridium difficile*, and *Klebsiella* spp., were not detected (median value 0 CFU/ m^2^, range 0–0 CFU/m^2^). Mycetes were however present, although at a relatively low level, with *Aspergillus* spp. representing the most prevalent (median value 0 CFU/m^2^, range 0–1263 CFU/ m^2^) followed by *Candida albicans* (median value 0 CFU/ m^2^, range 0–421 CFU/ m^2^). No significant differences were observed for any of the assayed pathogens at T0 in the two enrolled trains (*p* = 0.13).

After the implementation of PCHS sanitation as a substitute for chlorine disinfection (Fig. 3c–d), a remarkable decrease in pathogens was observed in both surfaces and air samples collected from the PCHS compared with the control train. Such a decrease was already evident at T1, 2 weeks after the introduction of PCHS, which remained steady and further intensified at the later times, leading to the virtual disappearance of the assayed surface pathogens at T5 (12 weeks after the introduction of PCHS).

In detail (Fig. 3c), at T1 (2 weeks after the introduction of PCHS sanitation), the total surface pathogens corresponded to 21,053 CFU/m^2^ (median value, range 0–139,789 CFU/m^2^) in the control train and to 8842 CFU/m^2^ (median value, range 0–26.105 CFU/m^2^) in the PCHS train (− 58%: *p* < 0.01). Of note, due to emergency indications, in the first 2 weeks of the study, chlorine disinfection was erroneously increased to four times per day for the control train; nevertheless, the once-a-day application of PCHS removed significantly more pathogens than this increased chlorine treatment.

At later times, the chlorine disinfection was applied once per day as originally scheduled and the difference between the control and PCHS trains resulted further evident. In fact, at T2 (corresponding to 4 weeks of PCHS application), surface pathogens amounted to 9263 CFU/m^2^ (median value, range 0–58,869 CFU/m^2^) in the control train and 3579 CFU/m^2^ (median value, range 0–25,689 CFU/m^2^) in the PCHS train (− 61%; *p* < 0.05). At T3 (6 weeks of PCHS), the surface pathogen load amounted to 11,158 CFU/m^2^ (median value, range 421–48,000 CFU/m^2^) in the control train and 2526 CFU/m^2^ (median value, range 0–9684 CFU/m^2^) in the PCHS train (− 77%; *p* < 0.001). At T4 (9 weeks), surface pathogens amounted to 10,526 CFU/m^2^ (median value, range 0–112,842 CFU/m^2^) in the control train vs. 1895 CFU/m^2^ (median value, range 0–17,263 CFU/m^2^) in the PCHS train (− 82%; *p* < 0.05). At T5, the final sampling time (12 weeks), surface pathogens were 2526 CFU/m^2^ (median value, range 0–84,832 CFU/m^2^) in the control train and 0 CFU/m^2^ (median value, range 0–11,368 CFU/m^2^) in the PCHS train (− 100%; *p* < 0.01).

The analysis of air filters yielded similar results (Fig. 3d). Filters were available for the analysis at times T0, T1, T2, and T4. In contrast to the situation at T0, when both trains had similar levels of pathogenic contamination, the values were significantly different in the two trains at later times. More specifically, in the control train, the pathogens entrapped in air filters amounted to 150,737 CFU/m^2^ at T1 (range 90,526–190,316 CFU/m^2^), 146,105 CFU/m^2^ at T2 (range 79,158–297,684 CFU/m^2^), and 292,632 CFU/m^2^ at T4 (range 170,526–444,632 CFU/m^2^). By contrast, in the PCHS train, the pathogens collected from air filters amounted to 91,789 CFU/m^2^ at T1 (range 18,526–147,789 CFU/m^2^), 84,842 CFU/m^2^ at T2 (range 66,947–121,263 CFU/m^2^), and 74,526 CFU/m^2^ at T4 (range 43,789–146,105 CFU/m^2^), evidencing stabilization of the total amount of potential pathogens in the air and a trend toward decrease, which was statistically significant at all tested timepoints, with a 39% decrease at T1, a 42% decrease at T3, and a 75% decrease at T4 (*p* < 0.001). Panels 3e–3f of Fig. 3 shows the results with the control values normalized to 100%, to better evidence the percentage decrease in the PCHS-train vs. the control one.

**Fig. 4:**
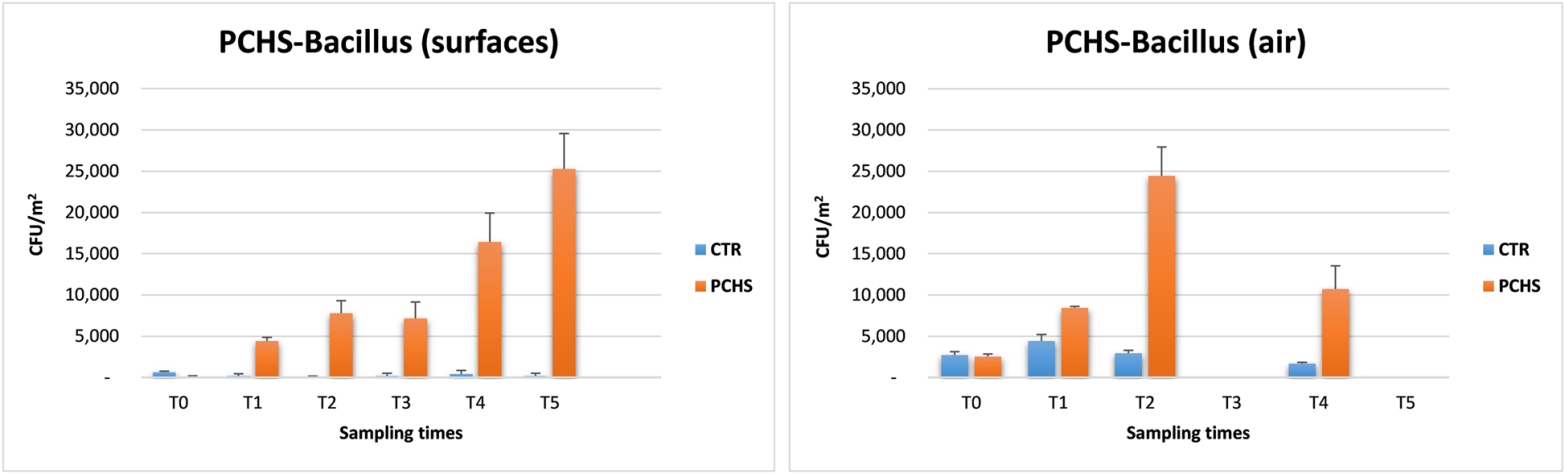
PCHS-*Bacillus* amount on surfaces and air of enrolled trains. *Bacillus* spp. CFUs were counted on Baird Parker RODAC agar plates. Results are expressed as median CFU value per m^2^ ± SD

As expected, the count of PCHS-*Bacillus* significantly increased in the PCHS train following the introduction of PCHS sanitation, whereas it remained stably low in the control train (Fig. 4). In the PCHS train, the amount of *Bacillus* ssp. increased from 0 CFU/m^2^ (median value, range 0–16,842 CFU/m^2^) at T0, to 4421 CFU/m^2^ (median value, range 0–21,895 CFU/m^2^), 7789 CFU/m^2^ (median value, range 0–29,053 CFU/m^2^), 7158 CFU/m^2^ (median value, range 842–229,895 CFU/m^2^), 16,421 CFU/m^2^ (median value, range 421–242,947 CFU/m^2^), and 25,263 CFU/m^2^ (median value, range 0–274,526 CFU/m^2^) at T1, T2, T3, T4 (*p* < 0.05), and T5 (*p* < 0.001), respectively. Similar results were found in the air filters, where the *Bacillus* count increased from 2526 CFU/m^2^ at T0 (median value, range 1263–5895 CFU/m^2^) to 8421 CFU/m^2^ (median value, range 8421–23,158 CFU/m^2^), 24,421 CFU/m^2^ (median value, range 9263–24,842 CFU/m^2^), and 10,737 CFU/m^2^ (median value, range 4632–17,263 CFU/m^2^) at T1, T2, and T4, respectively (*p* < 0.001 at all tested times) (Fig. 4). No increase in *Bacillus* count was observed in the control train at each time of sampling. Namely, *Bacillus* CFU median values on surfaces corresponded to 632 CFU/m^2^ (T0), 213 CFU/m^2^ (T1), 0 CFU/m^2^ (T2), 210 CFU/m^2^ (T3), 421 CFU/m^2^ (T4), and 211 CFU/m^2^ (T5). On air filters of the control train, *Bacillus* CFUs were 2737 CFU/m^2^ at T0, 2526 CFU/m^2^ at T1, 2947 CFU/m^2^ at T2, and 1684 CFU/m^2^ at T4.

### NGS characterization of trains’ microbiome

**Fig. 5:**
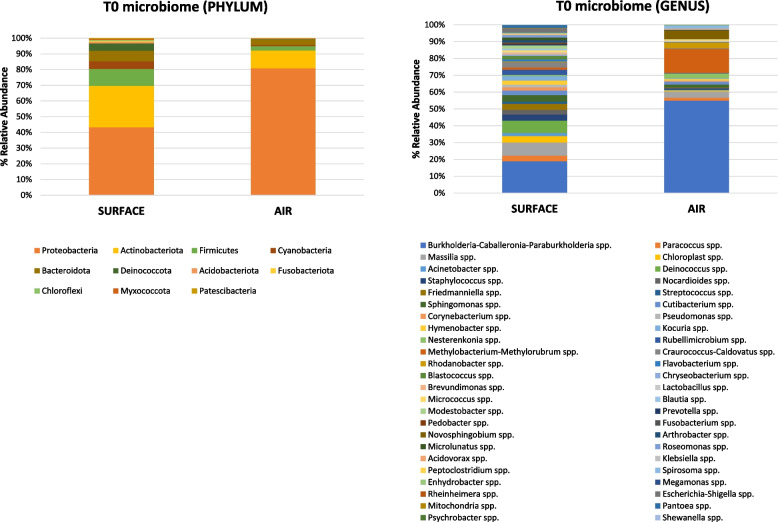
Surface and air microbiome composition at T0 in the enrolled trains. Characterization of the surface (floor) and air microbiomes by analyzing all the collected floor and air filter samples by 16S rRNA NGS. Percentage (%) relative abundances of phyla (left panel) and genera (right panel) are depicted. Results represent the mean relative abundance of all collected floor and air filter samples

Based on contamination levels detected in collected samples, surface and air microbiome characterization was performed on floor and air filter samples. Analysis of the taxonomy and community composition of the microbiome at T0, as evidenced by 16S rRNA NGS, showed the prevalence of *Proteobacteria* and *Actinobacteria* phyla in both trains, with relative abundance values of 42% and 25% in the floor, and 80% and 11% in the air, respectively. The same five most represented phyla were found in the surface and air samples, also showing a similar order of abundances, with *Firmicutes*, *Bacteroidota*, and *Cyanobacteria* representing respectively 10%, 7%, and 5% in surface samples and 3%, 3%, and 1% in the air. Overall, however, surface samples exhibited a higher amount and diversity in the less abundant phyla compared with air samples, with detectable *Deinococcota*,* Acidobacteria*, *Fusobacteria*, *Chloroflexi*, *Myxococcota*, and *Patescibacteria* phyla, whereas *Proteobacteria* and *Actinobacteria* together represented 91% of the total microbiome in air filters (Fig. 5).

At the genus level, *Burkholderia–Caballeronia–Parabulkolderia* spp. were the most abundant (11%) on surfaces, followed by *Massilia* (4.6%), *Deinococcus* (4.4%), *Chloroplast* (2.3%), *Sphingomonas* (2.3%), *Staphylococcus* (2.2%), *Friedmanniella* (2.1%), and *Paracoccus* (2%), which together comprised 31% of the total detected taxa. The human colonizers *Cutibacterium* (1.7%), *Corynebacterium* (1.1%), and *Streptococcus* (0.8%) were also detected, accompanied by potential human pathogens detected at low abundance levels, including *Escherichia–Shigella* spp. (up to 1.5%), *Acinetobacter* spp. (1.1%), *Pseudomonas* spp. (0.9%), and *Enterococcus* spp. (0.7%).

In air filters, similarly to surface microbiome, *Burkholderia–Caballeronia–Parabulkolderia* spp. were the most abundant (47%), followed by *Methylobacterium* (12.1%), *Novosphingobium* (4.4%), *Massilia* (3.2%), *Rhodanobacter* (2.8%), and *Nesterenkonia* (2.4%), which together comprised 72% of the total detected taxa. The genera *Paracoccus* (1.5%), *Spirosoma* (1.5%), *Cutibacterium* (1.4%), and *Sphingomonas* (1.4%) were also fairly represented, whereas *Micrococcus* (0.7%) and *Staphylococcus* (0.6%) were much less abundant compared to surfaces. The main phyla and genera detected in floor and air samples are reported in Suppl. Table 1.

**Fig. 6:**
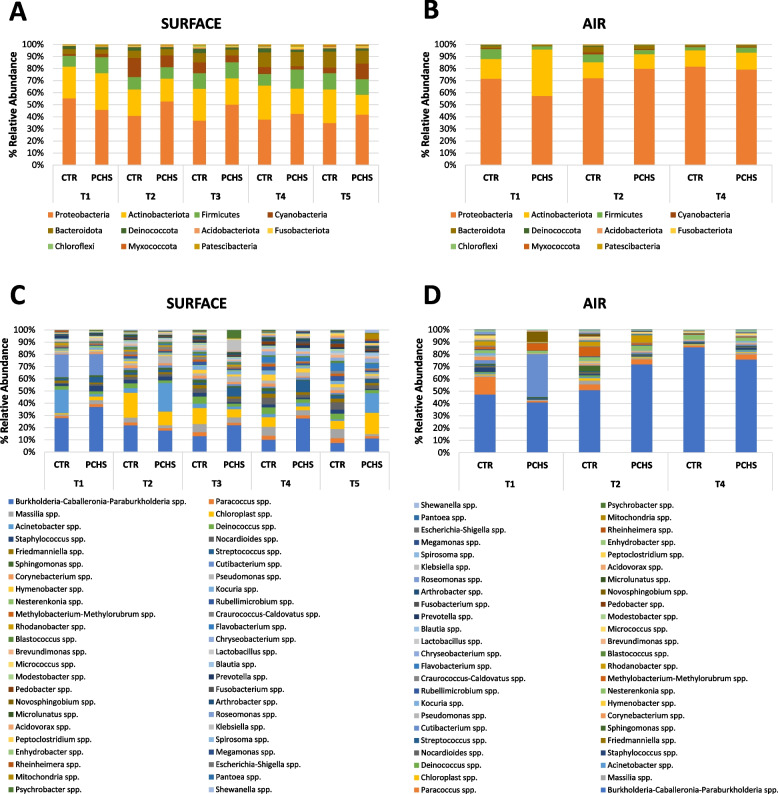
Surface and air microbiome composition in the enrolled trains after PCHS implementation (T1–T5). Characterization of the surface and air microbiomes by 16S rRNA NGS, and results are expressed as mean percentages of relative abundance. **a**, **b** Phylum composition and **c**, **d** genus composition of surface (floor) and air bacterial communities. CTR, control train; PCHS, PCHS-treated train. T1–T5, sampling timepoints

The results obtained in the surface and air samples collected at T1–T5, after PCHS implementation, indeed showed fair variability in the microbial community composition of both the control and PCHS trains, although the prevalence of *Proteobacteria* and *Actinobacteria* phyla was maintained throughout the whole study period, and the observed differences were not statistically significant at any time, whether between different sampling times in the same train or between control and PCHS train at a specific timepoint (Fig. 6a–b).

At the genus level (Fig. 6c–d), the results confirm the high fluctuations of relative abundance values of the different bacterial genera among the different sampling campaigns during the study time. Overall, however, the *Burkholderia–Caballeronia–Parabulkholderia* group was confirmed as the most prevalent at the different times in both trains, although showing great variations in its relative abundance. Similarly, *Acinetobacter*, *Cutibacterium*, *Chloroplast*, *Streptococcus*, and *Flavobacterium* genera were found to be abundant at all times, although with variation in amounts.

**Fig. 7:**
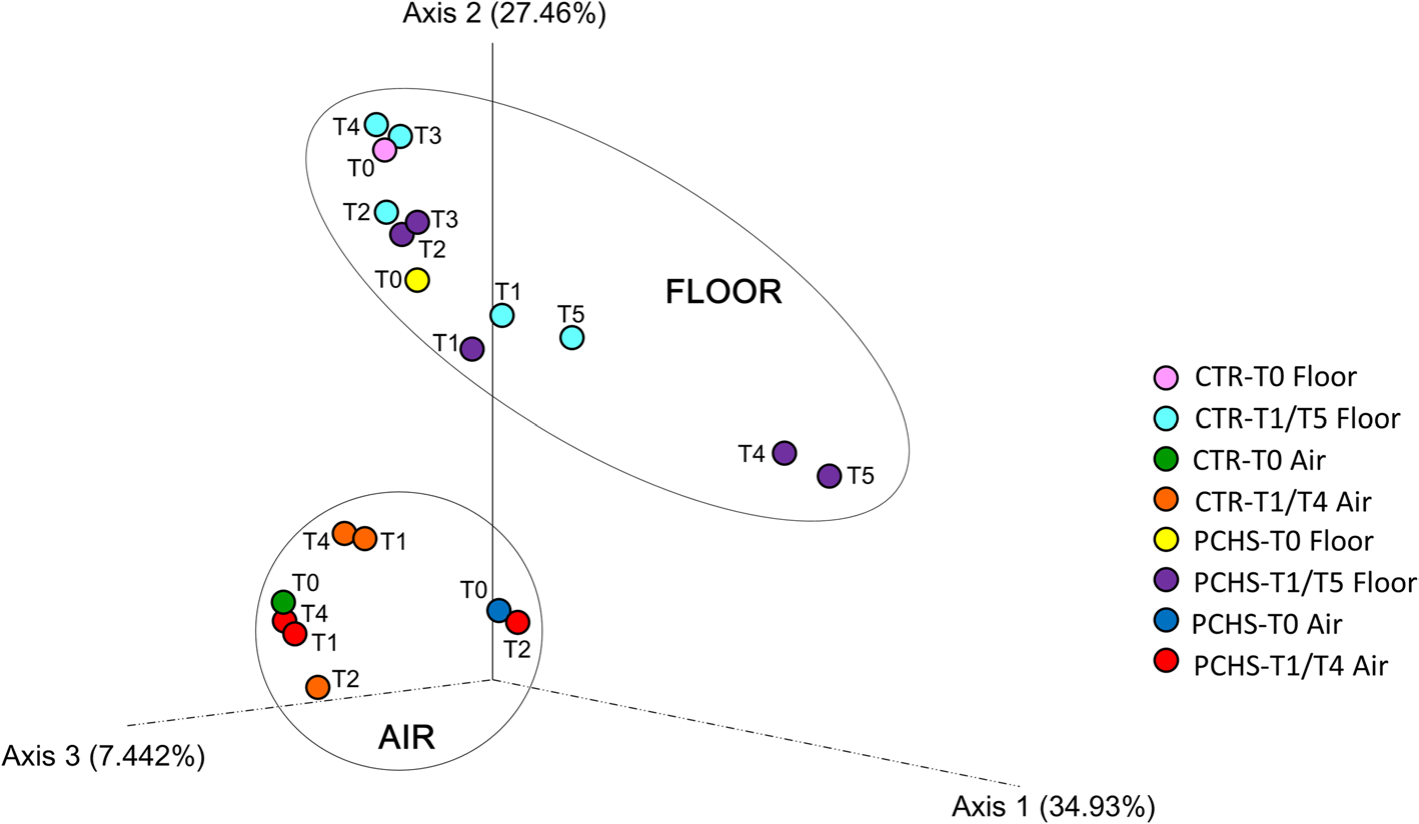
PERMANOVA analysis of collected samples showing differences in clustering of surface (floor) and air samples. Control train (CTR) and PCHS train (PCHS)

To detect any eventual similarity between the collected samples, NGS data were analyzed using the PERMANOVA assay, which accounts for both microbial composition and relative abundance. The results (Fig. 7) showed distinct clustering of surface (floor) and air samples and, again, with no evidence of any significant variation between control and PCHS trains at T0. At T1–T5 timepoints, no significant differences were observed in air filter samples, which continued to cluster very closely in both control and PCHS train. With regard to surface (floor) samples, no significant variances in microbial composition were detected at T1, T2, and T3 timepoints between trains; at later times (T4, T5), samples derived from the control train still clustered with the prior samples but those from the PCHS train grouped together and were distant from control samples, although the differences in diversity were not statistically significant.

**Fig. 8:**
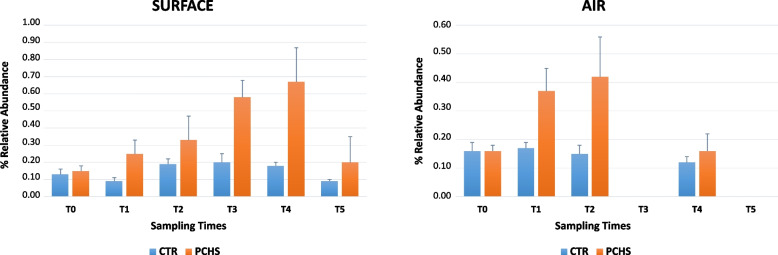
*Bacillus* spp. relative abundance in surfaces and air samples as detected by NGS analysis. The abundance of *Bacillus* spp. was evaluated in floor samples (left panel) as representative of train surfaces and in air samples (right panel) using 16S rRNA NGS. The results are expressed as mean values of percentage of relative abundance ± S.D. of all the samples collected at the indicated times (T0–T5)

Taken together, the NGS results did not indicate significant alterations in the microbiome composition of the PCHS train compared with the control, suggesting that PCHS implementation did not induce substantial gross variations on the whole microbiome but rather impacted on potential human pathogens, which were relatively less abundant in the whole train microbiome compared with the most prevalent environmental ones and thus better evidenced by direct CFU count rather than by NGS analysis. Regarding the *Bacillus* group, NGS analysis evidenced a low abundance of *Bacillus* spp. compared to the dominant bacterial genera, with relative abundance values < 1%. However, following PCHS implementation, a significant increase in *Bacillus* abundance was identifiable in the PCHS train compared with the control (*p* < 0.05) (Fig. 8), supporting the results obtained by direct *Bacillus* CFU count and culture-based detection methods.

Based on the small variations detected in the whole composition of the train microbial population, the train core microbiome was also analyzed, to assess in more detail any eventual impact of PCHS on the prevalence and abundance of the core taxa. The results showed the presence of 28 core genera, including the *Bacillus* genus (Suppl. Table 2). No significant variations in any of the prevalent core taxa were observed in PCHS vs. control train, consistent with the lack of significant differences in microbial composition detected by PERMANOVA analysis. Small significant differences were observed only in three less abundant genera, including *Roseomonas* and *Clostridium*, both slightly diminished in the PCHS-train compared to control (*p* = 0.034 and *p* = 0.007, respectively), and in the *Bacillus* genus, which was instead slightly increased (*p* = 0.035).

### Characterization of train resistome by qPCR

**Fig. 9:**
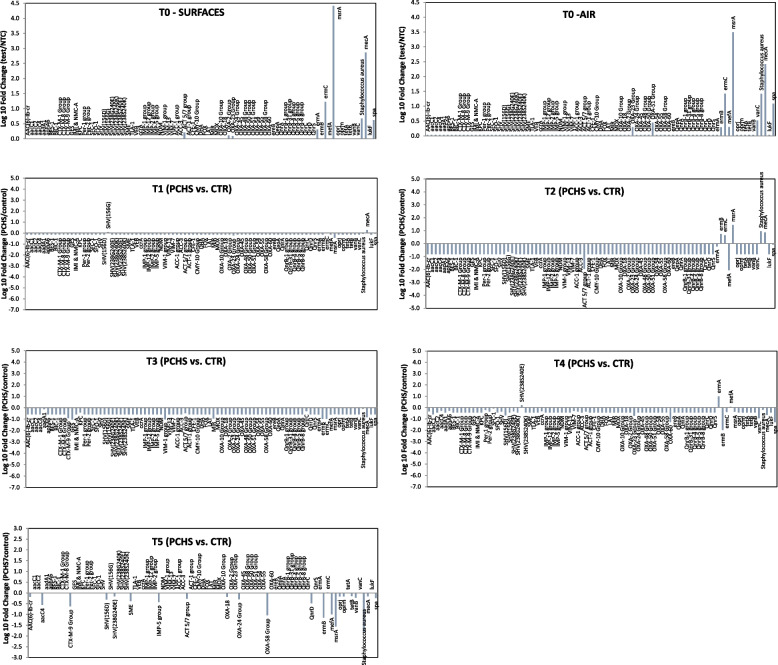
Characterization of the resistome of the train microbiome. Results obtained by qPCR microarray analysis performed in duplicate samples collected at T0, T1, T2, T3, T4, and T5 in PCHS and control trains. Original T0 resistome in surface samples and air filters are separately shown at the top of the graph. Cumulative results refer to surface and air at T1–T5 timepoints. Results are expressed as mean values ± S.D. of Log_10_ fold changes for each R gene when compared with controls

Besides their taxonomic composition, the surface and air train microbiomes were also characterized for their antimicrobial resistance AR gene content using a qPCR microarray to simultaneously identify and quantify 84 AR genes. The results showed that at T0 both train microbiomes harbored several R genes, conferring resistance against different antibiotic classes, including macrolides, methicillin, and class-C/class-D β-lactamases (Fig. 9). In particular, the most prevalent AR genes, in order of abundance, were *ermC*, *msrA*, and *mecA*, followed by lower but detectable levels of *OXA-2, OXA-23*, and *OXA-51* groups, *ACT 5/7* group, *ermA/ermB*, *mefA*, and *vanC* genes. The bacterial species *S. aureus* and its virulence gene *spa* (included in the resistome microarray) were also detected at a moderate level. Despite the original superimposable amounts of AR genes detected at T0 in the enrolled trains, at the subsequent times the AMR levels appeared substantially different in the two trains, as the control train roughly maintaining the AMR level found at T0 whereas in the PCHS train most of the AR genes decreased remarkably (Suppl. Table 3). Specifically, already at T1 sampling, almost all the AR genes detected at T0 decreased to undetectable levels in the PCHS-train compared with the control train samples (*p*_*c*_ < 0.01). At later times, the low level of AMR of the surface microbial population was maintained or even further decreased in the PCHS train compared with the chemically disinfected control train (Fig. 9).

### Analysis of SARS-CoV-2 presence

Due to the importance of assuring a SARS-CoV-2-free environment in public transportation at the time of the study, surface samples were also evaluated for the presence of SARS-CoV-2 RNA genome using a specifically targeted droplet digital PCR (ddPCR) able to detect even virus traces (namely 3 genome copies per sample). The results (Table 2) showed virus positivity in 21 out of the 136 total samples collected in the two trains (15.4%). At T0, two positive samples were detected in the control train (mean copy number 6.5 copies/sample) and one positive sample was detected in the train subsequently treated with PCHS (4.6 copies/sample), showing similar frequency of detection and virus load. By contrast, after PCHS introduction, significant differences were observed between the control and PCHS train. In detail, 13 further samples were found positive for the presence of SARS-CoV-2 RNA in control train (9.6%), with virus amounts varying from 6.8 to 27 copies per sample (corresponding to 340–1350 copies/mL), whereas five samples were found positive in PCHS train (3.7%), with copy numbers ranging from 4.6 to 9.4 copies per sample (corresponding to 230–470 copies/mL).

**Table 2:** Environmental SARS-CoV-2 contamination

Train	Time	N° positive/ total samples	Surface type	Load (^a^)
**CTR**	T0	2/136	Floor, Floor	4–9
	T1	2/136	Floor, Floor	6.8–10.5
	T2	2/136	Floor, Floor	8.5–12.4
	T3	3/136	Floor, Floor, Seat	10–21–18.5
	T4	3/136	Floor, Floor, Seat	24–10–27
	T5	3/136	Floor, Floor, Seat	14–26–17.6
**PCHS**	T0	1/136	Floor	4.6
	T1	0/136	-	-
	T2	2/136	Floor, Floor	9.4–4.6
	T3	0/136	-	-
	T4	1/136	Floor	4.8
	T5	2/136	Floor, Seat	5.2–6.1

^a^Virus load is expressed as genome copy number per sample (positivity: ≥ 3copies/sample in 20 µL); each sampling campaign consisted of 10 or 12 samples, depending on the availability of air filters

Overall, 17/21 positive samples derived from floors and 4/21 from seats, whereas no positivity was detected in samples from handrails, air filters, and doors. Of these, samples of twelve floor surfaces and three seats in the control train were positive, whereas samples of four floors and one seat in the PCHS train were found to be positive.

## Discussion

Mass transport environments, and specifically urban subways, are especially relevant to public health, since they represent distinct microbial environments with high occupant densities, diversities, and turnovers. During the COVID-19 pandemic, the particular importance of this aspect has emerged due to the high infectious risk linked with the continuous stream of human/human and human/BE microbial transmission. Consequently, government campaigns and actions were taken to reduce occupant density and to increase the subway environment disinfection aim to assure higher safety for traveling people. In particular, the widespread use of chemical disinfectants was mandatorily introduced to tackle the pandemic emergency [15] and such disinfection is largely still used for sanitation purposes in sanitary and non-sanitary environments, including in public transportation. However, most disinfectants have a temporary action [16], a profound impact on water and earth pollution [17], and the potential to induce increase of AMR [18, 19]. In an attempt to overcome such limitations, we recently set up and tested a low-impact method based on probiotic sanitation (PCHS, Probiotic Cleaning Hygiene System) in the hospital BE, showing that it can assure long-term hygienization without undesirable side effects of increased pollution or AMR. Indeed, its use compared with chemical disinfectants was associated with a stable (80%) drop in pathogens, accompanied by a 99.9% decrease of microbial AMR, and a 52% decrease in HAI incidence [16, 18, 20–26]. Since it also was proven to be safe for hospitalized patients [22] and to provide long-lasting decontamination from enveloped viruses [16, 27], we aimed to test PCHS applicability and effectiveness here through comparison with chemical disinfection for the sanitation of the subway environment during the COVID-19 emergency.

The study was set up as a pre–post and case–control study in two driverless trains of the Milan subway and lasted 12 weeks. Due to constraints related to COVID-19, it was not possible to completely replace the chemical disinfection with PCHS sanitation. Thus, a mixed regimen was applied, based on the results of preliminary tests to assess the compatibility between PCHS probiotics and chemical disinfectants. Namely, PCHS was applied 30 min after disinfection with ethanol or ammonium-based products and as a substitute for chlorine disinfectant.

The data collected during PCHS application evidenced significant differences in the number of pathogens in the control and PCHS trains, showing that probiotic sanitation resulted in rapid abatement of pathogens (− 58% in 2 weeks of use), leading to their virtual disappearance (− 100%) at the end of the trial (12 weeks of use), which is in contrast to the level of pathogens detected in the control train, which did not change significantly during the whole study period. This is despite the increased frequency of chlorine disinfection in the control train in the first 2 weeks of the study, which was performed four times per day, whereas PCHS was applied once a day. The decontamination ability of PCHS compared with chemical disinfectants was observed both on tested surfaces and in the air filters, starting from superimposable contamination levels and occupant density in the two enrolled trains, suggesting the genuine ability of PCHS to assure stable decontamination in contrast to the temporary action of disinfectants.

Interestingly, resistome analysis of the train microbiome showed that PCHS use was associated also with a drop in AR genes of up to 2-log compared with control train, confirming what has previously been observed in the sanitary environments treated by PCHS [20, 23, 25]. Interestingly, the data collected at T0 showed that the train microbiome harbored remarkable amounts of genes conferring resistance against beta-lactams (*ACT-5/7* group, *OXA-2*, and *OXA-23* groups), erythromycin and streptogramin (*ermA*, *ermC*, *msrA*), and methicillin (*mecA*), whereby the latter is associated with the presence of virulent *spa*-coding *S. aureus*, which was not evidenced by direct CFU count, confirming the higher sensitivity of molecular methods compared with culture-dependent methods [31]. The *spa*-expressing methicillin-resistant *S. aureus* (MRSA) strains are frequently detected in hospital environments [33], where they are often responsible for HAI onset; thus, our subway data underscore the spread of MRSA also in the general population in non-sanitary environments, confirming recent reports of antibiotic-resistant microorganisms in subways [34, 35], and highlighting the need for active surveillance of microbial communities in this environment.

To more deeply characterize the control and PCHS train microbiomes, the microbial population was also profiled by NGS to evidence the impact of PCHS on the whole train microbiome. At T0 and all subsequent timepoints, the five most prevalent phyla in both trains were *Proteobacteria*, *Actinobacteria*,* Firmicutes*, *Bacteroidota*, and *Cyanobacteria* in order of abundance, confirming previous data obtained in the subway environment [1, 3, 36]. The surface microbiome, compared with that of air, showed a higher abundance of less prevalent species of the *Deinococcota*,* Acidobacteria*, *Fusobacteria*, *Chloroflexi*, *Myxococcota*, and *Patescibacteria* phyla, whereas *Proteobacteria* and *Actinobacteria* represented nearly the total microbiome in air filters.

At the genus level, we detected colonizers of different ecological habitats, as also observed in other BE studies [37]. The most abundant group, in both surface and air samples, was *Burkholderia–Caballeronia–Parabulkholderia* (11% and 47%, respectively), a vast group of *Proteobacteria* that was mostly environmental was also detected in the human nasopharyngeal tract [38], and including beneficial species as well as animal and plant pathogens and around twenty potential human pathogens (of the *B. cepacia* group) [39]. The microbiome profiles in surface and air were composed differently regarding other prevalent genera. In particular, *Massilia*, *Deinococcus*,* Chloroplast*,* Sphingomonas*, and *Staphylococcus*, represented the five most abundant genera in surfaces after *Burkholderia* group (4.6%, 4.4%, 2.3%, 2.3%, 2.2%, respectively), whereas *Methylobacterium*,* Novosphingobium*,* Massilia*,* Rhodanobacter*, and *Nesterenkonia* were the top five genera in air filters (12.1%, 4.4%, 3.2%, 2.8%, and 2.4%, respectively). The surface/air diversity in microbial composition was consistently evidenced by PERMANOVA analysis, which showed different surface and air clusters, as also previously reported by others [1]. The passenger microbial input was also well represented in train microbiomes as several human colonizers were detected, although at lower abundance compared with predominantly environmental strains. *Staphylococcus*, *Cutibacterium*, *Corynebacterium*, *Streptococcus*,* Escherichia–Shigella*,* Acinetobacter*, *Pseudomonas*, and *Enterococcus* genera were observed at levels of abundance between 0.7 and 2.2%, similarly to what reported in previous studies [6, 36].

However, although the predominant phyla/genera maintained equal order of abundance during the study period, a remarkable variability in the percentage levels of individual abundances was observed in both trains with respect to the diverse detected bacterial groups; thus, no statistically significant alterations were detectable in the PCHS vs. control train. Consistent with this, also the 28 core taxa identified as the train core microbiome, did not vary significantly in PCHS-treated vs. control train, suggesting low or no impact of PCHS on the most prevalent environmental “signature” of the train microbiome. The core microbiome included also the *Bacillus* genus, as previously described [10]. Only three core taxa appeared significantly variated in the PCHS vs. control train, including the poorly represented *Bacillus*, *Roseomonas*, and *Clostridium* genera. Only *Bacillus* genus was slightly but significantly increased in the PCHS vs. control train (from 0.2 to 0.4% of relative abundance, *p* < 0.05), whereas *Roseomonas* and *Clostridium* (0.7% and 0.5% of relative abundance, respectively, in the control train) were both significantly diminished (0.3% relative abundance each; *p* < 0.05). This in contrast to the measured CFU counts and microarray data, which instead show a clear trend toward a decrease in pathogens and AMR in the PCHS vs. control train. This difference may be due to the low relative abundance of human pathogens and *Bacillus* spp. with respect to the whole train microbiome, which do not allow obtaining clearcut and significant percentage differences by comparison of such values. However, at least for *Bacillus* spp., a significant increase was detected in the PCHS train compared with the control, though the relative abundance of *Bacillus* remained under 1%. Compared with results obtained in the hospital environment, showing significant microbiome alterations after PCHS application [30, 31], the lack of significant differences observed in the subway microbiome may be linked to the different original composition of the train microbiome compared with that of the hospital. In fact, while the microbiome of the hospital environment is essentially of human origin [2, 40–42], the train microbiome appears mostly dominated by environmental species whose percentage fraction is scarcely influenced by the addition of *Bacillus*. By contrast, human pathogens, with their high nutritional needs, could be more effectively counteracted and inhibited by PCHS-*Bacillus* thanks to the mechanism of competitive exclusion.

Interestingly, the members of human microbiome we detected in the train microbiome mostly belong to the normal human skin and oral flora, as previously reported [1, 43], and these data, together with the AMR data, underscore the importance of active microbiological surveillance in mass transport environments, as a crowded environment favors horizontal transmission between microorganisms and human hosts. Furthermore, the microbes colonizing the environment can in turn contribute to the skin microbiome and resistome [8], and it is therefore important to control the environmental microbial communities, especially those living in spaces with a high density of human occupants, such as in mass transport.

The search of SARS-CoV-2 RNA evidenced its presence in both trains during the study period, although at low levels. RT-ddPCR results are not indicative of infectious virus; however, a significant decrease of positivity for virus RNA presence was detected in PCHS-train compared to chemically disinfected control train in the T1–T5 period, both in the percentage of positive samples (9.6 vs. 3.7%) and in the total viral copy number (340–1350 copies/ml vs. 230–470 copies/ml). Thus, the data suggest that PCHS sanitation could maintain the environment free of SARS-CoV-2 virus with an effectiveness comparable or superior to chemicals, as also previously proven in the hospital environment [27]. Floors were the most contaminated surfaces, as also reported for hospital environment. This, in light of the reported bacterial associations with SARS-CoV-2 presence both in humans [30] and in built environment [44], suggest that bacteria-virus synergy may play a role in the COVID-19 pandemic.

Limitations of the study include the duration of the study period, which was chosen in compliance with the current regulations, that may provide further pieces of information if longer or including different seasonal periods. Similarly, the analysis by NGS of a higher number of environmental samples could potentially better clarify any eventual significant variations upon the usage of chemical or PCHS sanitation, overcoming the lack of significance perhaps linked to the limited number of samples analysed in the present study (twenty total floor and air samples). Further work could explore the diversity of Milan subway microbial community and its impact on human health, since it has been reported that people living in developed countries have reduced microbiome diversity compared to non-urban environment and lifestyle [45]. Thus, Milan subway could be a useful field for future metacommunity studies on BE microbiomes and their impact on human population.

## Conclusions

In conclusion, this report details the first high-precision study of microbiome shaping in a subway environment following the use of chemical or biological probiotic-based sanitation as assessed using both culture-dependent and -independent methods. The results show that innovative eco-sustainable low-cost sanitation based on probiotic *Bacillus* could effectively replace the widespread use of chemicals, forgoing the risk of further exacerbating AMR and pollution. Also, the results indicated that the introduction of probiotic *Bacillus* strains do not modify substantially the whole environmental microbiome, leaving undisturbed the non-pathogenic environmental microbes and instead deeply impacting on the amount of potential pathogens.

Overall, the results also confirm that characterizing the microbial profiles of the environments populated by humans is increasingly important for the bio surveillance of AMR and pathogens, which may be used as an early indicator of outbreaks and, in parallel, would aid in the more rational design of public transport aiming to avoid the development of microbial reservoirs and thus preserve human health.

## Supplementary Information

**Additional file 1: Supplementary Table 1.** Main phyla and genera in air and floor samples at T0. Data are represented as mean of relative abundances.

**Additional file 2: Supplementary Table 2.** Train core microbiome: variations of core taxa in PCHS and control train compared to original composition (T0)(*).

**Additional file 3: Supplementary Table 3.** AR genes detected in train microbiomes at T0-T5 sampling timepoints (Log10 fold change values samples vs. NTC).

## Data Availability

The datasets generated and/or analysed during the current study are available in the Sequence Read Archive (SRA) repository, BioProject PRJNA891512.
