# Supplementary material for: Shaping the subway microbiome through probiotic-based sanitation during the COVID-19 emergency: a pre–post case–control study
Source: Microbiome. 2023 Mar 30;11:64. doi: 10.1186/s40168-023-01512-2 (PMC10060134; doi:10.1186/s40168-023-01512-2)
Supplement: Supplementary file 2 — Additional file 1: Supplementary Table 1. Main phyla and genera in air and floor samples at T0. Data are represented as mean of relative abundances. [file 40168_2023_1512_MOESM1_ESM.docx]

**Supplementary Table 1.** Main phyla and genera in air and floor samples at T0. Data are represented as mean of relative abundances.

|  | Air samples |  | Floor samples |
| --- | --- | --- | --- |
| Phylum | Abundance (%) | Phylum | Abundance (%) |
| *Proteobacteria* | 79.9 | *Proteobacteria* | 41.8 |
| *Actinobacteriota* | 11.2 | *Actinobacteriota* | 25.4 |
| *Bacteroidota* | 3.5 | *Firmicutes* | 10.4 |
| *Firmicutes* | 2.8 | *Bacteroidota* | 6.5 |
| *Cyanobacteria* | 0.7 | *Cyanobacteria* | 4.7 |
| *Acidobacteriota* | 0.2 | *Deinococcota* | 4.6 |
| *Deinococcota* | 0.2 | *Acidobacteriota* | 1.0 |
| *Chloroflexi* | 0.2 | *Chloroflexi* | 0.6 |
| *Myxococcota* | 0.1 | *Myxococcota* | 0.6 |
| *Patescibacteria* | 0.1 | *Patescibacteria* | 0.5 |
|  |  | *Fusobacteriota* | 0.4 |
|  |  |  |  |
| Genus | Abundance (%) | Genus | Abundance (%) |
| *Burkholderia-Caballeronia-Paraburkholderia spp.* | 46.6 | *Burkholderia-Caballeronia-Paraburkholderia spp.* | 11.3 |
| *Methylobacterium-Methylorubrum spp.* | 12.1 | *Massilia spp.* | 4.6 |
| *Novosphingobium spp.* | 4.4 | *Deinococcus spp.* | 4.4 |
| *Massilia spp.* | 3.2 | *Sphingomonas spp.* | 2.3 |
| *Rhodanobacter spp.* | 2.8 | *Chloroplast spp.* | 2.3 |
| *Nesterenkonia spp.* | 2.4 | *Staphylococcus spp.* | 2.2 |
| *Spirosoma spp.* | 1.5 | *Friedmanniella spp.* | 2.1 |
| *Paracoccus spp.* | 1.5 | *Paracoccus spp.* | 2.0 |
| *Cutibacterium spp.* | 1.4 | *Craurococcus-Caldovatus spp.* | 1.9 |
| *Sphingomonas spp.* | 1.4 | *Rubellimicrobium spp.* | 1.9 |
| *Hymenobacter spp.* | 0.8 | *Cutibacterium spp.* | 1.7 |
| *Micrococcus spp.* | 0.7 | *Nocardioides spp.* | 1.6 |
| *Staphylococcus spp.* | 0.6 | *Kocuria spp.* | 1.6 |
| *Chloroplast spp.* | 0.5 | *Hymenobacter spp.* | 1.5 |
| *Acidovorax spp.* | 0.5 | *Escherichia-Shigella spp.* | 1.5 |
| *Corynebacterium spp.* | 0.4 | *Blastococcus spp.* | 1.2 |
| *Craurococcus-Caldovatus spp.* | 0.4 | *Modestobacter spp.* | 1.2 |
| *Lactobacillus spp.* | 0.3 | *Acinetobacter spp.* | 1.1 |
| *Rubellimicrobium spp.* | 0.3 | *Corynebacterium spp.* | 1.1 |
| *Kocuria spp.* | 0.3 | *Pseudomonas spp.* | 0.9 |
