# Supplementary material for: Shaping the subway microbiome through probiotic-based sanitation during the COVID-19 emergency: a pre–post case–control study
Source: Microbiome. 2023 Mar 30;11:64. doi: 10.1186/s40168-023-01512-2 (PMC10060134; doi:10.1186/s40168-023-01512-2)
Supplement: Supplementary file 3 — Additional file 2: Supplementary Table 2. Train core microbiome: variations of core taxa in PCHS and control train compared to original composition (T0)(*). [file 40168_2023_1512_MOESM2_ESM.docx]

**Supplementary Table 2.** Train core microbiome: variations of core taxa in PCHS and control train compared to original composition (T0)(*)

| **Genus** | **Original** | **CTR** | **PCHS** | **PCHS vs. CTR (*p,* t test)** |
| --- | --- | --- | --- | --- |
| *Burkholderia-Caballeronia-Paraburkholderia* | 29.0 ± 23.3% | 25.9 ± 24.2% | 30.3 ± 22.3% | n.s. |
| *Methylobacterium-Methylorubrum* | 6.4 ± 11.1% | 1.5 ± 1.8% | 1.3 ± 1.6% | n.s. |
| *Massilia* | 3.9 ± 2.2% | 2.5 ± 1.5% | 1.8 ± 0.8% | n.s. |
| *Deinococcus* | 2.3 ± 2.5% | 2.0 ± 1.2% | 1.2 ± 0.8% | n.s. |
| *Sphingomonadaceae* | 2.0 ± 1.0% | 1.5 ± 0.8% | 1.3 ± 0.5% | n.s. |
| *Sphingomonas* | 1.8 ± 0.7% | 1.2 ± 1.0% | 0.6 ± 0.4% | n.s. |
| *Paracoccus* | 1.7 ± 0.7% | 3.2 ± 3.6% | 1.9 ± 1.0% | n.s. |
| *Microbacteriaceae* | 1.6 ± 0.7% | 1.7 ± 1.5% | 1.9 ± 1.1% | n.s. |
| *Cutibacterium* | 1.6 ± 1.2% | 2.7 ± 5.1% | 6.2 ± 10.9% | n.s. |
| *Nesterenkonia* | 1.4 ± 1.6% | 1.2 ± 1.1% | 1.3 ± 1.1% | n.s. |
| *Chloroplast* | 1.4 ± 1.4% | 4.0 ± 4.3% | 3.7 ± 4.2% | n.s. |
| *Staphylococcus* | 1.4 ± 0.9% | 1.6 ± 0.7% | 1.3 ± 0.9% | n.s. |
| *Hymenobacter* | 1.2 ± 0.6% | 1.1 ± 0.9% | 0.8 ± 0.6% | n.s. |
| *Craurococcus-Caldovatus* | 1.1 ± 0.9% | 0.9 ± 0.4% | 0.6 ± 0.4% | n.s. |
| *Rubellimicrobium* | 1.1 ± 0.9% | 1.1 ± 0.7% | 0.7 ± 0.3% | n.s. |
| *Nocardioides* | 1.0 ± 0.8% | 1.6 ± 1.2% | 1.1 ± 0.8% | n.s. |
| *Kocuria* | 0.9 ± 0.7% | 1.1 ± 0.7% | 0.9 ± 0.5% | n.s. |
| *Corynebacterium* | 0.8 ± 0.5% | 1.2 ± 0.6% | 0.7 ± 0.4% | n.s. |
| *Acinetobacter* | 0.7 ± 0.5% | 3.0 ± 5.1% | 4.2 ± 6.1% | n.s. |
| *Pseudomonas* | 0.6 ± 0.5% | 1.0 ± 0.4% | 1.5 ± 1.5% | n.s. |
| *Roseomonas* | 0.6 ± 0.4% | 0.7 ± 0.5% | 0.3 ± 0.1% | 0.034 |
| *Streptococcus* | 0.4 ± 0.4% | 0.9 ± 0.4% | 2.4 ± 2.2% | n.s. |
| *Comamonadaceae* | 0.4 ± 0.4% | 0.5 ± 0.5% | 0.4 ± 0.3% | n.s. |
| *Pseudonocardia* | 0.4 ± 0.3% | 0.4 ± 0.2% | 0.3 ± 0.2% | n.s. |
| *Lactobacillus* | 0.4 ± 0.1% | 0.7 ± 0.4% | 0.4 ± 0.4% | n.s. |
| *Clostridium* | 0.4 ± 0.2% | 0.5 ± 0.2% | 0.3 ± 0.1% | 0.007 |
| *Enhydrobacter* | 0.2 ± 0.1% | 0.4 ± 0.3% | 0.3 ± 0.2% | n.s. |
| *Bacillus* | 0.2 ± 0.0% | 0.2 ± 0.1% | 0.4 ± 0.1% | 0.035 |

(*) Results are expressed as mean of relative abundances ± S.D.; n.s., not significant.
