# Supplementary material for: Shaping the subway microbiome through probiotic-based sanitation during the COVID-19 emergency: a pre–post case–control study
Source: Microbiome. 2023 Mar 30;11:64. doi: 10.1186/s40168-023-01512-2 (PMC10060134; doi:10.1186/s40168-023-01512-2)
Supplement: Supplementary file 4 — Additional file 3: Supplementary Table 3. AR genes detected in train microbiomes at T0-T5 sampling timepoints (Log10 fold change values samples vs. NTC). [file 40168_2023_1512_MOESM3_ESM.docx]

**Suppl. Table 3.** AR genes detected in train microbiomes at T0-T5 sampling timepoints (*)

|  | T0 | | T1 | | T2 | | T3 | | T4 | | T5 | |
| --- | --- | --- | --- | --- | --- | --- | --- | --- | --- | --- | --- | --- |
| AR gene | **CTR** | **PCHS** | **CTR** | **PCHS** | **CTR** | **PCHS** | **CTR** | **PCHS** | **CTR** | **PCHS** | **CTR** | **PCHS** |
| *AAC(6)-Ib-cr* | 0 | 0 | 0 | 0 | 0 | 0 | 0 | 0 | 0 | 0 | 0.182 | 0 |
| *aacC1* | 0 | 0 | 0 | 0 | 0 | 0 | 0 | 0 | 0 | 0 | 0 | 0 |
| *aacC2* | 0 | 0 | 0 | 0 | 0 | 0 | 0 | 0 | 0.129 | 0 | 0 | 0 |
| *aacC4* | 0 | 0 | 0 | 0 | 0 | 0 | 0 | 0 | 0 | 0 | 0.552 | 0 |
| *aadA1* | 0 | 0 | 0 | 0 | 0 | 0 | 0 | 0 | 0 | 0 | 0 | 0 |
| *aphA6* | 0 | 0 | 0 | 0 | 0 | 0 | 0 | 0 | 0 | 0 | 0 | 0 |
| *BES-1* | 0 | 0 | 0 | 0 | 0 | 0 | 0 | 0 | 0 | 0 | 0 | 0 |
| *BIC-1* | 0 | 0 | 0 | 0 | 0 | 0 | 0 | 0 | 0 | 0 | 0 | 0 |
| *CTX-M-1 Group* | 0 | 0 | 0 | 0 | 0 | 0 | 0 | 0 | 0 | 0 | 0 | 0 |
| *CTX-M-8 Group* | 0 | 0 | 0 | 0 | 0 | 0 | 0 | 0 | 0 | 0 | 0 | 0 |
| *CTX-M-9 Group* | 0 | 0 | 0 | 0 | 0 | 0 | 0.335 | 0 | 0 | 0 | 0.622 | 0 |
| *GES* | 0 | 0 | 0 | 0 | 0 | 0 | 0.460 | 0 | 0 | 0 | 0 | 0 |
| *IMI & NMC-A* | 0 | 0 | 0 | 0 | 0 | 0 | 0 | 0 | 0 | 0 | 0 | 0 |
| *KPC* | 0 | 0 | 0 | 0 | 0 | 0 | 0 | 0 | 0 | 0 | 0 | 0 |
| *Per-1 group* | 0 | 0 | 0 | 0 | 0 | 0 | 0 | 0 | 0 | 0 | 0 | 0 |
| *Per-2 group* | 0 | 0 | 0 | 0 | 0 | 0 | 0 | 0 | 0.446 | 0.076 | 0 | 0 |
| *SFC-1* | 0 | 0 | 0 | 0 | 0 | 0 | 0 | 0 | 0 | 0 | 0 | 0 |
| *SFO-1* | 0 | 0 | 0 | 0 | 0 | 0 | 0 | 0 | 0 | 0 | 0 | 0 |
| *SHV* | 0 | 0 | 0 | 0 | 0 | 0 | 0 | 0 | 0 | 0 | 0 | 0 |
| *SHV(156D)* | 0 | 0 | 0 | 0 | 0 | 0 | 0 | 0 | 0.202 | 0 | 0.305 | 0 |
| *SHV(156G)* | 0 | 0 | 0 | 0.212 | 0 | 0 | 0 | 0 | 0 | 0 | 0 | 0 |
| *SHV(238G240E)* | 0 | 0 | 0 | 0 | 0 | 0 | 0 | 0 | 0 | 0.001 | 0.159 | 0 |
| *SHV(238G240K)* | 0 | 0 | 0 | 0 | 0 | 0 | 0 | 0 | 0 | 0 | 0 | 0 |
| *SHV(238S240E)* | 0 | 0 | 0 | 0 | 0 | 0 | 0 | 0 | 0 | 0 | 0 | 0 |
| *SHV(238S240K)* | 0 | 0 | 0 | 0 | 0 | 0 | 0 | 0 | 0 | 0 | 0 | 0 |
| *SME* | 0 | 0 | 0 | 0 | 0 | 0 | 0 | 0 | 0 | 0 | 0.376 | 0 |
| *TLA-1* | 0 | 0 | 0 | 0 | 0 | 0 | 0 | 0 | 0 | 0 | 0 | 0 |
| *VEB* | 0 | 0 | 0 | 0 | 0 | 0 | 0 | 0 | 0 | 0 | 0 | 0 |
| *ccrA* | 0 | 0 | 0 | 0 | 0 | 0 | 0 | 0 | 0 | 0 | 0 | 0 |
| *IMP-1 group* | 0 | 0 | 0 | 0 | 0 | 0 | 0 | 0 | 0 | 0 | 0 | 0 |
| *IMP-12 group* | 0 | 0 | 0 | 0 | 0 | 0 | 0 | 0 | 0 | 0 | 0 | 0 |
| *IMP-2 group* | 0 | 0 | 0 | 0 | 0 | 0 | 0 | 0 | 0 | 0 | 0 | 0 |
| *IMP-5 group* | 0 | 0 | 0 | 0 | 0 | 0 | 0 | 0 | 0 | 0 | 0.428 | 0 |
| *NDM* | 0 | 0 | 0 | 0 | 0 | 0 | 0 | 0 | 0 | 0 | 0 | 0 |
| *VIM-1 group* | 0 | 0 | 0 | 0 | 0 | 0 | 0.445 | 0 | 0 | 0 | 0 | 0 |
| *VIM-13* | 0 | 0 | 0 | 0 | 0 | 0 | 0 | 0 | 0 | 0 | 0 | 0 |
| *VIM-7* | 0 | 0 | 0 | 0 | 0 | 0 | 0 | 0 | 0 | 0 | 0 | 0 |
| *ACC-1 group* | 0 | 0 | 0 | 0 | 0 | 0 | 0 | 0 | 0 | 0 | 0 | 0 |
| *ACC-3* | 0 | 0 | 0 | 0 | 0 | 0 | 0 | 0 | 0 | 0 | 0 | 0 |
| *ACT 5/7 group* | 0.215 | 0.175 | 0 | 0 | 0.930 | 0 | 0 | 0 | 0 | 0 | 0.269 | 0 |
| *ACT-1 group* | 0 | 0 | 0 | 0 | 0 | 0 | 0 | 0 | 0 | 0 | 0 | 0 |
| *CFE-1* | 0 | 0 | 0 | 0 | 0 | 0 | 0 | 0 | 0 | 0 | 0 | 0 |
| *CMY-10 Group* | 0 | 0 | 0 | 0 | 0 | 0 | 0 | 0 | 0 | 0 | 0 | 0 |
| *DHA* | 0 | 0 | 0 | 0 | 0 | 0 | 0 | 0 | 0 | 0 | 0 | 0 |
| *FOX* | 0 | 0 | 0 | 0 | 0 | 0 | 0 | 0 | 0 | 0 | 0 | 0 |
| *LAT* | 0 | 0 | 0 | 0 | 0 | 0 | 0 | 0 | 0 | 0 | 0 | 0 |
| *MIR* | 0 | 0 | 0 | 0 | 0 | 0 | 0.371 | 0 | 0 | 0 | 0 | 0 |
| *MOX* | 0 | 0 | 0 | 0 | 0 | 0 | 0 | 0 | 0 | 0 | 0 | 0 |
| *OXA-10 Group* | 0 | 0 | 0 | 0 | 0 | 0 | 0 | 0 | 0 | 0 | 0 | 0 |
| *OXA-18* | 0 | 0 | 0 | 0 | 0 | 0 | 0 | 0 | 0 | 0 | 0.188 | 0 |
| *OXA-2 Group* | 0.121 | 0.092 | 0 | 0 | 0 | 0 | 0 | 0 | 0 | 0 | 0 | 0 |
| *OXA-23 Group* | 0.216 | 0.277 | 0.398 | 0.348 | 0 | 0 | 0 | 0 | 0.324 | 0 | 0 | 0 |
| *OXA-24 Group* | 0 | 0 | 0 | 0 | 0 | 0 | 0 | 0 | 0 | 0 | 0.431 | 0.118 |
| *OXA-45* | 0 | 0 | 0 | 0 | 0 | 0 | 0 | 0 | 0 | 0 | 0 | 0 |
| *OXA-48 Group* | 0 | 0 | 0 | 0 | 0 | 0 | 0 | 0 | 0 | 0 | 0 | 0 |
| *OXA-50 Group* | 0 | 0 | 0 | 0 | 0 | 0 | 0 | 0 | 0 | 0 | 0 | 0 |
| *OXA-51 Group* | 0.333 | 0 | 0 | 0 | 0 | 0 | 0 | 0 | 0.238 | 0 | 0 | 0 |
| *OXA-54* | 0 | 0 | 0 | 0 | 0 | 0 | 0 | 0 | 0 | 0 | 0 | 0 |
| *OXA-55* | 0 | 0 | 0 | 0 | 0 | 0 | 0 | 0 | 0 | 0 | 0 | 0 |
| *OXA-58 Group* | 0 | 0 | 0 | 0 | 0 | 0 | 0 | 0 | 0 | 0 | 1.041 | 0 |
| *OXA-60* | 0 | 0 | 0 | 0 | 0 | 0 | 0 | 0 | 0.676 | 0 | 0 | 0 |
| *ereB* | 0 | 0 | 0 | 0 | 0 | 0 | 0 | 0 | 0 | 0 | 0 | 0 |
| *QepA* | 0 | 0 | 0 | 0 | 0 | 0 | 0 | 0 | 0 | 0 | 0 | 0 |
| *QnrA* | 0 | 0 | 0 | 0 | 0 | 0 | 0 | 0 | 0 | 0 | 0 | 0 |
| *QnrB-1 group* | 0 | 0 | 0 | 0 | 0 | 0 | 0 | 0 | 0 | 0 | 0 | 0 |
| *QnrB-31 group* | 0 | 0 | 0 | 0 | 0 | 0 | 0 | 0 | 0 | 0 | 0 | 0 |
| *QnrB-4 group* | 0 | 0 | 0 | 0 | 0 | 0 | 0 | 0 | 0 | 0 | 0 | 0 |
| *QnrB-5 group* | 0 | 0 | 0 | 0 | 0 | 0 | 0 | 0 | 0 | 0 | 0 | 0 |
| *QnrB-8 group* | 0 | 0 | 0 | 0 | 0 | 0 | 0 | 0 | 0 | 0 | 0 | 0 |
| *QnrC* | 0 | 0 | 0 | 0 | 0 | 0 | 0 | 0 | 0 | 0 | 0 | 0 |
| *QnrD* | 0 | 0 | 0 | 0 | 0 | 0 | 0 | 0 | 0 | 0 | 0.483 | 0 |
| *QnrS* | 0 | 0 | 0 | 0 | 0 | 0 | 0 | 0 | 0 | 0 | 0 | 0 |
| *ermA* | 0.198 | 0.256 | 0 | 0 | 0 | 0 | 0.125 | 0.098 | 0 | 1.194 | 0 | 0 |
| *ermB* | 0.210 | 0.237 | 0 | 0 | 0.481 | 1.084 | 1.013 | 0.607 | 1.396 | 0.143 | 2.163 | 0.911 |
| *ermC* | 1.135 | 1.121 | 0.717 | 0.628 | 0.480 | 0.956 | 1.036 | 0.648 | 0.300 | 0.156 | 0.888 | 0.819 |
| *mefA* | 0.213 | 0.209 | 0 | 0 | 1.298 | 0.277 | 0.423 | 0.101 | 0.120 | 0.154 | 0.991 | 0 |
| *msrA* | 3.825 | 4.068 | 3.734 | 3.414 | 1.952 | 2.582 | 2.887 | 2.890 | 1.485 | 1.037 | 4.156 | 2.339 |
| *oprj* | 0 | 0 | 0 | 0 | 0 | 0 | 0 | 0 | 0 | 0 | 0.169 | 0 |
| *oprm* | 0 | 0 | 0 | 0 | 0 | 0 | 0 | 0 | 0 | 0 | 0.161 | 0 |
| *tetA* | 0 | 0 | 0 | 0 | 0 | 0 | 0 | 0 | 0 | 0 | 0 | 0 |
| *tetB* | 0 | 0 | 0 | 0 | 0 | 0 | 0 | 0 | 0 | 0 | 0.150 | 0 |
| *vanB* | 0 | 0 | 0 | 0 | 0 | 0 | 0 | 0 | 0 | 0 | 0.218 | 0 |
| *vanC* | 0.397 | 0.498 | 0 | 0 | 0 | 0 | 0 | 0 | 0.406 | 0 | 0 | 0 |
| *S. aureus* | 1.130 | 1.560 | 1.020 | 1.005 | 0.536 | 1.027 | 0.209 | 0.108 | 0.347 | 0.172 | 2.830 | 1.214 |
| *mecA* | 1.948 | 2.584 | 1.260 | 1.494 | 0.738 | 1.070 | 1.900 | 1.041 | 0.552 | 0.477 | 0.786 | 0.147 |
| *lukF* | 0 | 0 | 0 | 0 | 0 | 0 | 0 | 0 | 0 | 0 | 0 | 0 |
| *spa* | 0.857 | 0.718 | 0.712 | 0.799 | 0.120 | 0.114 | 0 | 0 | 0.089 | 0 | 1.217 | 0 |

(*) Results are expressed as Log_10_ fold change values of samples *vs.* NTC.
